# Medication adherence perspectives in haemodialysis patients: a qualitative study

**DOI:** 10.1186/s12882-017-0583-9

**Published:** 2017-05-22

**Authors:** Saurav Ghimire, Ronald L. Castelino, Matthew D. Jose, Syed Tabish R. Zaidi

**Affiliations:** 1 Unit for Medication Outcomes Research and Education (UMORE), Pharmacy, School of Medicine, Faculty of Health, University of Tasmania, Hobart, 7001 Australia; 2 Sydney Nursing School, University of Sydney, Sydney, Australia; 3 Blacktown Hospital, Western Sydney Local Health District, Sydney, Australia; 4 School of Medicine, Faculty of Health, University of Tasmania, Hobart, Australia; 5 Department of Nephrology, Royal Hobart Hospital, Hobart, Australia

**Keywords:** End-stage kidney disease, Haemodialysis, Medication adherence, Patients’ perspectives, Qualitative study

## Abstract

**Background:**

End-stage kidney disease patients undergoing haemodialysis are prescribed with multiple complex regimens and are predisposed to high risk of medication nonadherence. The aims of this study were to explore factors associated with medication adherence, and, to examine the differential perspectives on medication-taking behaviour shown by adherent and nonadherent haemodialysis patients.

**Methods:**

A qualitative exploratory design was used. One-on-one semi-structured interviews were conducted with 30 haemodialysis patients at the outpatient dialysis facility in Hobart, Australia. Patient self-reported adherence was measured using 4-item Morisky Green Levine scale. Interview transcripts were thematically analysed and mapped against the World Health Organization (WHO) determinants of medication adherence.

**Results:**

Participants were 44–84 years old, and were prescribed with 4–19 medications daily. More than half of the participants were nonadherent to their medications based on self-reported measure (56.7%, *n* = 17). Themes mapped against WHO adherence model comprised of patient-related (knowledge, awareness, attitude, self-efficacy, action control, and facilitation); health system/ healthcare team related (quality of interaction, and mistrust and collateral arrangements); therapy-related (physical characteristics of medicines, packaging, and side effects); condition-related (symptom severity); and social/ economic factors (access to medicines, and relative affordability).

**Conclusions:**

Patients expressed a number of concerns that led to nonadherence behaviour. Many of the issues identified were patient-related and potentially modifiable by using psycho-educational or cognitive-behavioural interventions. Healthcare professionals should be more vigilant towards identifying these concerns to address adherence issues. Future research should be aimed at understanding healthcare professionals’ perceptions and practices of assessing medication adherence in dialysis patients that may guide intervention to resolve this significant issue of medication nonadherence.

**Electronic supplementary material:**

The online version of this article (doi:10.1186/s12882-017-0583-9) contains supplementary material, which is available to authorized users.

## Background

An estimated 2.6 million people worldwide received dialysis treatment for End-stage kidney disease (ESKD) in 2010, and a two-fold increase is expected by 2030 [1]. Developing a new molecule into a medicine for clinical use costs about $2.6 billion [2], whereas the cost of treating complications from medication nonadherence averages about $100 billion a year [3]. Medication nonadherence is highly prevalent in ESKD patients undergoing haemodialysis with an average prevalence rate of 52.5% [4]. The consequences of medication nonadherence are detrimental and costly in haemodialysis patients [4–6], as these patients have increased burden of co-existing illness and prescribed with multiple complex regimens [7–10]. Younger age, higher comorbidities, frequent hospitalizations, poly-pharmacy, and high pill burden have been consistently reported as predictors of low medication adherence in haemodialysis patients [4, 11–13]. These adherence predictors have been mainly identified through quantitative methods. However, these methods are less capable of exploring patients’ perspectives on medication-taking behaviour and the challenges they face while adhering to their prescribed regimens [14]. There are limited number of studies that have examined patients’ perspectives on renal failure, treatment adherence, dietary constraints, and phosphate binding medications [14–16]. To date, little is known about haemodialysis patients’ perceptions regarding their prescribed regimen and the factors influencing their medication-taking behaviour. Understanding patients’ perspectives can help identify potentially modifiable factors such as patients’ intention to adhere, beliefs about medicines, features about treatment regimens, experiences of side effects, and provision of support mechanism required to facilitate adherence [17]. As such, we aimed to qualitatively explore factors associated with medication adherence, and examine the differential perspectives on medication-taking behaviour shown by haemodialysis patients.

## Methods

### Study design

A qualitative exploratory design was used. The consolidated criteria for reporting qualitative research (COREQ) guideline [18] was followed during the conduct and reporting of the study (Additional file 1: Appendix 1). Ethics approval was granted by the Tasmanian Health and Medical Human Research Ethics Committee (H0014506). Written informed consent was obtained from all the participants.

### Research team and reflexivity

Interviews were conducted by a pharmacist researcher (SG). The interviewer was external to the study site, and both the participants and the interviewer were unknown to each other before the study. The study aims and professional status of the interviewer were discussed with the participants prior to conducting the interviews.

### Participants

All adult (≥ 18 years), English speaking patients, undergoing haemodialysis at the outpatient dialysis unit in Hobart, Australia were eligible to participate. Participant recruitment was sought from patients who had earlier participated in a cross-sectional study [19] that investigated association between medication regimen complexity and medication adherence in haemodialysis patients. This study had a good response rate of above 75%, with 53 haemodialysis patients completing the study. These patients were re-invited for participation for the qualitative interview. Thirty haemodialysis patients consented for the qualitative interview whereas nonparticipation by the rest was mainly due to lack of interest, fatigue or inconvenience.

### Data collection and analysis

One-to-one interviews were held during the dialysis session. All interviews were conducted by SG between February and June 2015, using the interview guide (Additional file 1: Appendix 2), and the median interview duration was 17.5 min (range, 6–41 min). All interview sessions were audio-recorded and transcribed verbatim; patients were not remunerated for their participation. Data on socio-demographic and clinical characteristics were obtained during interviews and by reviewing medical records. Adherence was determined by self-reports using the 4-item Morisky Green Levine scale [20]. Patients with a Morisky score of zero were considered adherent and those scoring 1–4 were considered nonadherent, based on similar studies assessing self-reported adherence in haemodialysis patients [19, 21].

Interview transcripts were thematically analysed [22]. Transcripts were repeatedly read for familiarization and data immersion. Two investigators (SG and STRZ) independently coded and reviewed the first five transcripts to ensure concordance was reached. Remaining transcripts were coded by SG and the final themes were agreed upon by both SG and STRZ. The analysis was iterative during data collection and carried out following each interview. Data saturation was assumed after 18 interviews however, all participants who consented for the study were interviewed. Themes generated were mapped against the World Health Organization (WHO) determinants of medication adherence that included patient-related-, health system/ healthcare team related-, therapy-related-, condition-related-, and social/ economic factors [23]. Patient-related factors within the WHO model was further sub-divided into aspects such as knowledge, awareness, attitude, self-efficacy, action control, and facilitation; based on adherence support taxonomy of behaviour change techniques [24].

## Results

Table 1 shows the study characteristics of the participants. The median age was 71 years (range, 44–87 years), and the patients were taking 4–19 medications daily. More than half of the participants were nonadherent to their medications based on self-reported measure (56.7%, *n* = 17). The major themes classified according to WHO determinants of adherence is presented below. The exemplar quotes for each theme is provided in Table 2. Full compilation of quotations is supplied as Additional file 1: Appendix 3. Please note the following abbreviation for the section below: P = patient (with a number to indicate the interview sequence for example, P5 is the fifth interviewed patient).

**Table 1:** Characteristics of study participants (*n* = 30)

Variables	Number (%)
Age, in years	69.6 ± 11.0
40–59	5 (16.7)
60–79	18 (60.0)
≥ 80	7 (23.3)
Gender, male	23 (76.7)
Marital status, married	17 (56.7)
Living with family	18 (60.0)
Level of education, ≥ high school	24 (80.0)
Smoking history, non-smoker	24 (80.0)
Number of medicines prescribed	11.4 ± 4.3
1–5	4 (13.3)
6–10	7 (23.3)
≥ 11	19 (63.3)
Daily pill burden	16.0 ± 6.1
1–9	4 (13.3)
10–19	15 (50.0)
≥ 20	11 (36.7)
Years on dialysis	4.1 ± 4.1
< 1	6 (20.0)
1–5	17 (56.7)
≥ 6	7 (23.3)
Hospitalization (past 1 year)^b^	22 (73.3)
Dialysis session missed^b^	5 (16.7)
Diabetes	7 (23.3)
Hypertension	17 (56.7)
Cardiovascular disease	16 (53.3)
Adherence to medication^a^	
Adherent	13 (43.3)
Nonadherent	17 (56.7)

For continuous variables, Mean ± SD; for categorical variables, numbers with percentages in parentheses

^a^Adherence to medication was based on self-reported measure using 4-item Morisky Green Levine scale. Patients scoring zero were considered adherent

^b^At least one event of hospitalization or dialysis session missed in past 1 year prior to the month of data collection

**Table 2:** Determinants of medication adherence in haemodialysis patients

Themes based on WHO taxonomy	Exemplar quotes
Patient-related^a^	
Knowledge and belief about medicines	
- Lack of understanding about medicines	*“Well, I just don’t know what some of them are for.”* (P1, male, 53 years, PSR NAD)
	*“I don’t know what’s really important and… if you missed [medication] once or twice it wouldn’t matter, I’ve no idea.”* (P5, female, 58 years, PSR NAD)
	*“As far vitamins are no much point for me because it all gets dialysed out of here [pointing to the dialysis machine].”* (P8, male, 71 years, PSR NAD)
- Lack of benefit	*“I don’t know if they doing any good? […] I thought well, you know, I am taking all this in the morning, um… are they doing any good? I don’t know.”* (P5, female, 58 years, PSR NAD)
- Safety concerns	*“There’s one medicine that is a statin which I’m very unhappy about. It’s Atorvastatin. And, I’m unhappy about that… because they… they, ah, studies have shown that there are lots of side effects of that.”* (P6, female, 74 years, PSR NAD)
- Relative importance	*“I think blood pressure one is important. Yes, I think that is important to keep my blood pressure down…”* (P6, female, 74 years, PSR NAD)
- Perceived need	*“There’s something to do with my kidney and that. […] it’s not working very well. If I started not taking them, I could for been… you know in trouble. They all they are for a reason. Yeah.”* (P15, male, 78 years, PSR AD)
- Perceived effectiveness	*“I put myself on that [medicine] because I didn’t have any arthritis or anything before I started [dialysis] and all of a sudden my fingers going, and I put it on that now for a month and it stopped the pain…”* (P12, female, 80 years, PSR AD)
Awareness and attitude	
- Motivation to live	*“I don’t know how much longer I got to live. But I want to get up to 80. If I become 80, that will be the longest lived in all our family. And if I make 80… I’m the champion.”* (P15, male, 78 years, PSR AD)
- Positive attitude	*“I got to take them as they keep me healthy. And I don’t have a problem with it.”* (P21, male, 84 years, PSR AD)
- General dislike	*“I don’t like the fact that I need to take them… Not happy about taking medications but the alternatives not good.”* (P13, female, 63 years, PSR NAD)
Self-efficacy	
- Disruption to daily routine	*“Well it’s in the morning and night, I’m just used to doing that. It’s the middle one I have to take care of… I take it at night. Take two at night instead of three, spreading three during the day, which the doctor asked me to try, because it might be more effective. I haven’t yet succeeded.”* (P8, male, 71 years, PSR NAD)
- Inconvenience during travel	*“People don’t make it difficult for me, but it’s the fact that I’ve, I travel, I like to travel of course make it difficult, because I’ve got to take all the stuffs with me, organize something every day or whatever. Yes, traveling.”* (P6, female, 74 years, PSR NAD)
- Accustomed regimen	*“I got all these medications every day, morning, evening, night. So, I never forget it, now.”* (P15, male, 78 years, PSR AD)
	*“I’ve been taking it for a long time and it’s just natural.”* (P27, male, 79 years, PSR AD)
- Unaccustomed regimen	*“I’m supposed to take a medicine for my [restless leg], but I keep forgetting… So, um, I’ve only been told this few days ago and I haven’t got used to it, to taking it.”* (P8, male, 71 years, PSR NAD)
Action control	
- Forgetfulness	*“Well, I think that I’m much more, I don’t know, forgetful then I used to be, I can’t think this clearly… seems I pick but I don’t. Um. Remembering to take it. I think that’s the biggest thing.”* (P6, female, 74 years, PSR NAD)
- Stimuli or cues for action	*“I have a little pill boxes, it holds all morning, noon and night… I just take whatever is required during dinner, or at meal in the night.”* (P15, male, 78 years, PSR AD)
- Visual allocation of pills	*“I’ve got them [medicines] in the kitchen table, so I can’t forget.”* (P10, female, 53 years, PSR AD)
- Association with meals	*“If I don’t have lunch, I don’t remember my medicines, always. Lunch is sort of tied to the medicines. So, if I wouldn’t eat, I wouldn’t take the medicines so regularly, I think.”* (P6, female, 74 years, PSR NAD)
Facilitation	
- Role of support	*“My wife makes sure I take them... she helps. She gets all medicines ready, tablets ready… she does all, mostly.”* (P27, male, 79 years, PSR AD)
	*“Some medicines make me dizzy. It is a problem. Especially when I get no support at home. Coz my husband, he works at night, and I got to be careful. Coz I got no support at home.”* (P7, female, 65 years, PSR NAD)
Health system/ HCT-related	
Quality of interaction with HCT	
- One-way communication	*“[Asking Dr. about the need of so many medicines…] I saw doctor at the clinic last time and he said, “No, they are all good”. He went through one by one [medicines] and no, that’s good, you need that, you need that, so…”* (P7, female, 65 years, PSR NAD)
- Lack of engagement	*“[Consultations are] never very long usually, you know. Just checks the figures, just look at your blood figures and everything’s ok and you know.”* (P2, male, 61 years, PSR NAD)
- Lack of time	*“I really need to speak to the pharmacist. Um, but they’re very busy, but I will, I must speak to, I want to know what every medicines, especially 12 medicines in the morning are for.”* (P5, female, 58 years, PSR NAD)
- Support from HCT	*“It’s always great with my GP. I’ve been going to him for 15 years and we’re quite informal and he’s very helpful and if I complained about what these things, he investigates them properly.”* (P11, male, 84 years, PSR AD)
	*“You know, just, give all your tablets to the chemist and he’ll sort them out. Makes it so much easier. He puts them in [Webster-Pak] … for 2 weeks and you got a just twist and pop a tablets… so I don’t need to worry about what one of this, one of this, anymore.”* (P16, male, 65 years, PSR AD)
Mistrust and collateral arrangements	
- Pressure to hide	*“I forgot to say [doctor] about it [not taking phosphate binders]. Because, I think what they will gonna tell me is, I have to take it. I’m frightened obvious the doctor’s gonna say, which they probably will, because it’s very important, the phosphate, I know that.”* (P5, female, 58 years, PSR NAD)
- Being a good patient	*“I don’t. I don’t know I take it because I’ve been told to take it, and I do that. But I don’t take it very seriously. And if I miss it, I don’t get panic, so.”* (P8, male, 71 years, PSR NAD)
- Personal control of treatment	*“I discuss it with myself. Or, I go to [doctor] who gets upset because I decide to take more than what I’m prescribed. Like the Sifrol, it wasn’t holding, so I lifted the [dose] up to two. And checked it [in the internet] and it was okay to do that and then she [doctor] got most upset because she said it effects the kidney, and I said well they’re pretty shot already, and she said they can always get worse.”* (P8, male, 71 years, PSR NAD)
- Trust in HCT	*“I take my medicines. They give me the right thing, so I just take them. Except when I’m allergic to.”* (P10, female, 53 years, PSR AD)
	*“I keep taking them until my doctor takes me out of it. I just take the dose that’s on the charts I got.”* (P25, male, 72 years, PSR AD)
Therapy-related	
Physical characteristics of medicines	
- Pill size	*“I’ve got the one [medicine], got to cut it half, I’ve got a cut five or six in half so I’ve got half for in the morning and half at night.”* (P9, female, 63 years, PSR NAD)
- Palatability	*“Some of them, as soon as you get them on the tongue…I swear it, dissolves straight away and it tastes disgusting! First thing in the morning they, oh! […] Just bitter, you know, one of them.”* (P5, female, 58 years, PSR NAD)
Medicine packaging	*“One I have very hard to get it out. A little capsule, that for pain. Yeah. Very hard to put out. The capsules are completely crushed by the time it gets out of its thing! That’s the only problem.”* (P11, male, 84 years, PSR AD)
Side effects of medicines	*“Sometimes they work, sometimes really make me sick. Makes me dizzy. It’s a bit stronger. I don’t take them. If they are not too strong, I’ll take them… but if they make me dizzy, I don’t.”* (P7, female, 65 years, PSR NAD)
	*“I don’t like taking them, the [antibiotics], they give me toilet all the time.”* (P29, male, 65 years, PSR NAD)
Social/ economic	
Access to medicines	
- Acquiring script	*“I’m taking a lot of pain tablets at the moment… I was taking patches, but you can’t get more than a month’s supply. So, that means going back on doctors, and when I get out of here [dialysis], I don’t want… to get to the doctors on my days off [from dialysis], so I’m just taking Panadol and Panadol with Codeine. But, is not really enough, to be honest.”* (P2, male, 61 years, PSR NAD)
- Clinic and pharmacy location	*“Because I live out of town… and about 40 min from the chemist, just kind of be aware how many more medicines I’ve got, it’s nothing worse than running out and having to drive especially for that, yeah.”* (P3, male, 44 years, PSR NAD)
	*“Some of the scripts you can’t get from [local pharmacy]. So, I’ve had issues actually getting them in the past… When my doctor goes on holidays, I can’t acquire a script without doing it a 100 km drive. [Dialysis staffs] refused to help me, and the doctors refused to give scripts over the phone. I can’t acquire a script over the phone…”* (P1, male, 53 years, PSR NAD)
Relative affordability	*“Well, they’re quite expensive! So they do affect me, the cost. I don’t have a health care card. I’ve to pay the full subsidised price… I’ve retired and so I’m living of an allocated pension from my superannuation.”* (P4, male, 56 years, PSR NAD)
	*“The only thing that worries me is, coz I’m in a wheel chair and I need to get to the hospital to get the scripts, it means for $ 30 to get in the taxi to go in there and pick up the script or I drive my mobility scooter all the way in there, which means 2 hours and an hour of each waiting to pick them up.”* (P2, male, 61 years, PSR NAD)
Condition-related	
Symptom severity	*“Have you seen me 12 months ago, I am on a 100% better [condition] after this year but last year and a year before, no, I didn’t really think I’m gonna make it. Not even everybody else also gonna make it either.”* (P12, female, 80 years, PSR AD)
	*“I don’t notice any [improvement] from my medications, whatsoever.”* (P1, male, 53 years, PSR NAD)

*Abbreviations:* AD, Adherent; NAD, nonadherent; BP, blood pressure, HCT, healthcare team; PSR, patient self-reports

^a^Patient-related factors further classified based on adherence support taxonomy by de Bruin et al., 2010 [24]

### Theme 1: Patient-related factors

#### Knowledge and belief about medicines

Patients assigned variable importance to their prescribed medicines and it appeared that the patients who were less informed of the purpose of their medicines see little for taking them regularly (P1; P5). This lack of understanding also led to the misconception that some of their medicines get washed out during dialysis and would remain ineffective (P8). Such misconceptions triggered doubts about their necessity, which led to prioritizing medication due to lack of benefit (P1; P5), and relative importance given to some medicines (P6), thus encouraging nonadherence behaviour. Furthermore, some patients acquired nonadherent behaviour as they expressed safety concerns about their medications (P5; P6). On the other hand, patients having better understanding about their disease process had higher perceived need (P11; P15; P16) and developed perceived effectiveness (P12) of their medication therapy and were therefore adherent.

#### Awareness and attitude towards medicines

Being aware of the consequences of nonadherence such as deterioration of medical condition and in rare cases, fear of death was found to be a motivator to be adherent. Motivated patients desiring to live longer (P12; P15; P20; P21; P25) and those expressing positive attitude towards taking medicines (P10; P11; P15; P21; P24; P28) were thus found to be adherent. On the contrary, a patient who was not motivated to overcome the general dislike of taking medicine was likely to demonstrate a nonadherent behaviour (P13).

#### Self-efficacy

Patient’s ability to manage their medication in different situations also influenced their medication-taking behaviour. Disruption to daily routine, particularly the midday dosing frequency, was identified as a practical barrier to medication adherence. This was pertinent in patients expressing personal preferences of taking medications at their conveniences (P8), or in those prioritizing important life events of the day besides taking medicines (P18). Also, some participants accentuated that carrying medicines and remembering to take them was inconvenient during their travel and outdoor activities (P3; P6). Whereas, patients accustomed to their regimen after following a routine for a relatively longer span of time were found to be adherent (P15; P16; P20; P21; P27; P30) while, a patient who was unaccustomed with his recent changes in medication regimen had a tendency to forget and was more likely to be nonadherent (P8).

#### Action control

Patient’s capacity to control medication intake as planned was also influencing medication adherence. Participants expressed forgetfulness as an excuse for not taking medication and gave an impression that nonadherence was unintentional (P6; P8; P14). Adherent patients, though, made their circumstances favourable for taking medicines by using stimuli such as pill boxes (P15; P18; P25) or by visibly allocating their pills (P10; P12). Furthermore, some patients related their meals and medicines together by stating that skipping meals during the day might end-up with them not taking their medicines (P5; P6).

#### Facilitation

Patients who were influenced and reinforced by their family members (P12; P15; P21; P27) were better adhering to their medications whereas, patients expressing lack of support from their family members (P7) or those who lived alone (P2) were found to be nonadherent to their medications.

### Theme 2: Health system/ healthcare team related factors

#### Quality of interaction with healthcare team

Few patients expressed dissatisfaction from their interaction and engagement with the healthcare team and were likely to demonstrate nonadherent behaviour. Some of the issues raised by these patients include, one-sided communication by their physician (P7); lack of engagement during consultation visits (P2; P4); and lack of time for medication counselling (P5). Some patients avoided discussing adherence related issues with their doctor as they had a preconceived notion about what their doctors would say. This might have occurred due to a prior unpleasant interaction with their doctor. For instance, a participant remembered an occasion where doctor showed less empathy towards her unresolved symptoms despite taking medicines (P5). On the other hand, patients expressing satisfaction from their interaction and engagement with the healthcare professionals tend to be adherent to their medications (P11; P16).

#### Mistrust and collateral arrangements

A general lack of trust on healthcare team particularly towards medical profession was observed in some patients. Those who perceived that their concerns towards medications will not be attended to by their doctors preferred either hiding their concerns (P5) or portrayed themselves as a good patient (P6; P8). Dissatisfaction and mistrust, following unpleasant interaction with physicians, may have further aggravated patients in making parallel or collateral arrangements for themselves by surpassing physicians’ decision and recommendation regarding their medications. Patients thus exerted a sense of personal control over their treatment (P2; P7; P8). In contrast, patients who were having a satisfying and trustworthy relationship with their doctors seemed to have followed the prescribed instructions in a relatively unopposed fashion (P10; P11; P15; P20; P25).

### Theme 3: Therapy-related factors

#### Physical characteristics of medicines

Physical characteristics of medicines were considered to hinder adherence in some patients. Pharmaceutical make-ups such as size of pills especially the larger ones (for e.g. phosphate binders) were considered difficult to swallow (P9; P10). Also, few patients complained about palatability of medicines to be a nuisance when they have to be taken early in the morning (P5; P13; P22).

#### Side effects of medicines

Some patients complained about side effects such as dizziness, nausea, vomiting and diarrhoea after taking their medicines (P5; P7; P29). They experimented on their own by skipping doses and observing if the symptoms persisted. When patients were convinced that their past experiences of untoward symptoms were the results of taking their medicines (P5; P7; P29), they would prefer avoiding those medicines such that they would not suffer from similar adverse effects in the future.

#### Packaging of medicines

A patient considered one of his medicine packaging to be non-user-friendly (P11), posing it to be a practical barrier that impeded adherence.

### Theme 4: Social/ economic factors

#### Access to medicine

Medicines in Australia are supplied at a subsidised cost through the Pharmaceutical Benefits Scheme (PBS). However, access to some specialised medicines routinely used in dialysis patients (such as Lanthanum, Erythropoietin, Cinacalcet etc.) is restricted to specialised pharmacies and hospital based clinicians. This particularly becomes an issue while acquiring scripts when the patients are living far away from the major town or cities where they are offered limited access to professional medical services (P1; P3). Besides, patients are also constrained with filling their prescription to not more than a month’s supply under the PBS, which makes a frequent consultation visit for acquiring scripts inconvenient in patients having dialysis fatigue and chronic incapacitation (P2; P23). This results into medicine shortage and the patients are compelled to choose readily available over-the-counter medicines that may not always be an effective alternative (P2). Besides acquiring scripts, the clinic and pharmacy location also posed a limitation to access medicines in patients living in remote areas (P1; P3).

#### Relative affordability

The relative affordability of medicines due to cost or financial constraints was another factor that impede access to medicines and ultimately contributed to nonadherence. Some of the haemodialysis patients were over the retirement age, and mainly lived on disability support pension or superannuation disability benefits (P2; P4). Although patients were getting medicines in a highly subsidized price through the PBS, these tend to cover only the partial costs for prescription medicines. However, due to the complexity of disease treatment and associated symptom burden, patients often required additional over-the-counter medicines including multi-vitamin preparations, vitamin D, iron and mineral supplements, pain medicines etc., which are not covered by the benefit schemes and patients needed them to pay out-of-pocket. The relative affordability of these medicines when considering the cost of acquiring script, transportation, and medicine costs itself restrained patients to access their medicines (P2; P4; P5).

### Theme 5. Condition-related factors

#### Symptom severity

Severity of symptoms had an influence on patient’s risk perception, importance of following treatment regimen, and the priority they placed on medication adherence. A patient who observed decreased symptom severity over time was found to be adherent with her prescribed regimen (P12). However, another patient who didn’t see any improvement of his health condition was found to be nonadherent (P1).

## Discussion

This qualitative study explored factors associated with medication adherence in haemodialysis patients, and examined their perceptions on medication-taking behaviour. A dissonance of perceptions with respect to adherence behaviour was observed between adherent and nonadherent patients. Factors mostly influencing medication-taking behaviour were patient-related. Some of the factors identified corroborated with past findings such as safety concerns of medicines [10, 14, 25], disruption to daily routine [14], forgetfulness [10, 25], use of reminders [14, 16], and role of social support [16, 21].

Knowledge and beliefs about medicines was an essential patient-specific factor potentially impeding adherence behaviour. Prioritization of medicines due to poor understanding, perceived necessity and concerns was a major reason for nonadherence. These findings in relation to patients’ beliefs regarding their medication can be further studied through various behavioural model on adherence (for e.g. Medication Adherence Model, Health Belief Model, Theory of Planned Behaviour) [26–28]. Belief components such as necessity and concerns can be specifically targeted by utilizing tool such as Beliefs about Medicines Questionnaires (BMQ), particularly the Specific-Necessity and Specific-Concerns scale [29]. Patients’ belief about necessity and concerns related to medicine can be overcome through psycho-educational interventions [30]. Thus, our study re-emphasizes on the need for providing medication-related information to combat patient ignorance about medications in haemodialysis patients [14, 31].

Patients reporting poor interaction with healthcare providers displayed a compromised adherence behaviour. In particular, patients were less satisfied from the consultations that lack inquiry about their experiences of taking medicines and any adverse effects they might be suffering. Although patients reporting medication-related symptoms to their physician is less frequent, physicians not necessarily always respond to them, even if they were reported [32, 33]. Suboptimal patient-physician interaction may lead to patients losing trust on physicians’ recommendations and hiding their concerns while trying to be a good patient [10]. This may also lead to patients making collateral arrangement for their medications to exert a flawed sense of control over their treatment, resulting in nonadherence. Thus, it is extremely important for the healthcare professionals to routinely instigate dialogs on medication issues with patients and encourage them to volunteer such information if they were not being asked for during consultations [32].

Socio-economic factors such as access to medicines and its affordability also raised concerns that hindered adherence. Access to prescribed medicines and professional medical services gradually declines when moving away from metropolitan cities through rural and remote locations [34]. Although our study site was located in the metropolitan city, some patients visiting the dialysis centre lived in rural areas and were required to travel to the city where they could access to professional advice for acquiring prescriptions or repeat them from the pharmacy. Though eligible patients benefitted from the government subsidy schemes for the cost reductions in prescription medicines [35], the large financial burden accumulated from the number of prescription and non-prescription medicines, the cost of acquiring scripts, transportation, and out-of-pocket payments annulled the cost benefits from the subsidy in haemodialysis patients. Medicine affordability can be much more challenging for patients in developing countries where public healthcare system does not guarantee subsidy of prescription medicines and the patients generally does not subscribe to health coverage schemes [36].

This study finding have both clinical and research implications. As dialysis patients, coupled with comorbid illness and dialysis-associated complications continually demands high pill burden for treatment, we tend to lose considerations on how polypharmacy, regimen complexity, and adherence issues should be addressed. As such, this study provides a subjective account of patients’ concerns that may lead to nonadherence. Healthcare professionals may routinely instigate dialogs and encourage patients to volunteer information concerning their current medicines, readiness to start new therapy, changes with dose or dosage requirements, and side-effects or safety concerns they might be dealing with. Any transitioning of medication therapy may be facilitated by providing personalized education by capitalising on the need and importance of taking medicines. Improving access to professional medical and pharmaceutical services and developing dialysis centre-based intervention programs focussing on the psycho-educational support may be effective. The same framework may be utilized in research settings to develop behavioural and educational interventions for examining patient concerns associated with medication adherence.

Study limitations need a mention. This is a single-centred study that may limit the generalizability of the findings. Interviews were conducted with English speaking patients only, thus, the findings may not be generalizable to non-English speaking patients. Although the participants were interviewed in an outpatient setting of a tertiary care metropolitan hospital, some of the patients came from rural areas driven by access limited healthcare services and support mechanisms. Hence, the access barrier gained attention in our themes, which may only be true for patients living in rural areas [25]. As interviews were conducted during dialysis sessions, patients may have been hesitant in responding freely while sharing their experiences. Furthermore, interviews for research purpose may have facilitated social desirability response [37], though it was unlikely as a wide-ranging viewpoints were expressed. Despite limitations, we used a purposive sampling method to identify participants of different demographic characteristics, and showing different medication-taking behaviour that best represented the perspectives of patients regarding the phenomenon under study.

## Conclusions

Haemodialysis patients expressed a number of concerns that led to nonadherence behaviour. Many of the issues identified were patient-related and potentially modifiable by using psycho-educational or cognitive-behavioural interventions. Healthcare professionals should be more vigilant towards identifying these concerns to address adherence issues. Future research should be aimed at understanding healthcare professionals’ perceptions and practices of assessing medication adherence in dialysis patients that may guide intervention to resolve this significant issue of medication nonadherence.

## Additional file

Additional file 1 Appendix 1 Consolidated criteria for reporting qualitative studies (COREQ): 32-item checklist. Appendix 2 Interview guide. Appendix 3. Summary of interpretation of themes with exemplar quotes. (DOCX 32 kb)

## Abbreviations

BMQ: Beliefs about Medicines Questionnaires
COREQ: Consolidated Criteria for Reporting Qualitative Research
ESKD: End-Stage Kidney Disease
PBS: Pharmaceutical Benefits Scheme
WHO: World Health Organization
