# Supplementary material for: Medication adherence perspectives in haemodialysis patients: a qualitative study
Source: BMC Nephrol. 2017 May 22;18:167. doi: 10.1186/s12882-017-0583-9 (PMC5440949; doi:10.1186/s12882-017-0583-9)
Supplement: Additional file 1 — Appendix 1 Consolidated criteria for reporting qualitative studies (COREQ): 32-item checklist. Appendix 2 Interview guide. Appendix 3. Summary of interpretation of themes with exemplar quotes. (DOCX 32 kb) [file 12882_2017_583_MOESM1_ESM.docx]

**Appendix 1.** Consolidated criteria for reporting qualitative studies (COREQ): 32-item checklist

| **No. Item** | **Guide questions/ description** | **Reported in section/ remarks** |
| --- | --- | --- |
| **Domain 1: Research team and reflexivity** | | |
| *Personal Characteristics* |  |  |
| 1. Interviewer/ facilitator | Which author/s conducted the interview or focus group? | Research team and reflexivity |
| 2. Credentials | What were the researcher’s credentials? E.g. PhD, MD | PhD |
| 3. Occupation | What was their occupation at the time of the study? | Research team and reflexivity |
| 4. Gender | Was the researcher male or female? | Male |
| 5. Experience and training | What experience or training did the researcher have? | SG was trained for pharmacists Home Medicines Review on medication history taking and collating medication-related information and provided one-on-one coaching to interview complex patients by the hospital pharmacist. |
| *Relationship with participants* |  |  |
| 6. Relationship established | Was a relationship established prior to study commencement? | Research team and reflexivity |
| 7. Participant knowledge of the interviewer | What did the participants know about the researcher? e.g. personal goals, reasons for doing the research | Research team and reflexivity.  Data collection. |
| 8. Interviewer  characteristics | What characteristics were reported about the interviewer/ facilitator? e.g. Bias, assumptions, reasons and interests in the research topic | SG discussed prior literature on  Medication nonadherence in haemodialysis patients, and how little is known about the patients’ perspectives in outpatient dialysis setting. |
| **Domain 2: Study design** |  |  |
| *Theoretical framework* |  |  |
| 9. Methodological  orientation and Theory | What methodological orientation was stated to underpin the study? e.g.  grounded theory, discourse analysis, ethnography, phenomenology, content analysis | Data collection and analysis |
| *Participant selection* |  |  |
| 10. Sampling | How were participants selected? e.g. purposive, convenience, consecutive,  snowball | Participants |
| 11. Method of approach | How were participants approached? e.g. face-to-face, telephone, mail, email | Participants |
| 12. Sample size | How many participants were in the study? | Participants |
| 13. Non-participation | How many people refused to participate or dropped out? Reasons? | Participants |
| *Setting* |  |  |
| 14. Setting of data  collection | Where was the data collected? e.g. home, clinic, workplace | Data collection and analysis |
| 15. Presence of nonparticipants | Was anyone else present besides the participants and researchers? | No |
| 16. Description of sample | What are the important characteristics of the sample? e.g. demographic data, date | Participants |
| *Data collection* |  |  |
| 17. Interview guide | Were questions, prompts, guides provided by the authors? Was it pilot tested? | Data collection and analysis. Appendix 2. |
| 18. Repeat interviews | Were repeat interviews carried out? If yes, how many? | Not conducted |
| 19. Audio/ visual recording | Did the research use audio or visual recording to collect the data? | Data collection and analysis |
| 20. Field notes | Were field notes made during and/or after the interview or focus group? | Data collection and analysis |
| 21. Duration | What was the duration of the interviews or focus group? | Data collection and analysis |
| 22. Data saturation | Was data saturation discussed? | Data collection and analysis |
| 23. Transcripts returned | Were transcripts returned to participants for comment and/ or correction? | No |
| **Domain 3: Analysis and findings** | | |
| *Data analysis* |  |  |
| 24. Number of data coders | How many data coders coded the data? | Data collection and analysis |
| 25. Description of the  coding tree | Did authors provide a description of the coding tree? | No. Intermediate documentation is available upon request. |
| 26. Derivation of themes | Were themes identified in advance or derived from the data? | Data collection and analysis |
| 27. Software | What software, if applicable, was used to manage the data? | N/A |
| 28. Participant checking | Did participants provide feedback on the findings? | No |
| *Reporting* |  |  |
| 29. Quotations presented | Were participant quotations presented to illustrate the themes/ findings? Was each quotation identified? e.g. participant number | Results. Table 2.  Appendix 3. |
| 30. Data and findings  consistent | Was there consistency between the data presented and the findings? | Yes |
| 31. Clarity of major themes | Were major themes clearly presented in the findings? | Results |
| 32. Clarity of minor themes | Is there a description of diverse cases or discussion of minor themes? | Results |

Developed from: Tong A, Sainsbury P, Craig J. Consolidated criteria for reporting qualitative research (COREQ): a 32-item checklist for interviews and focus groups. *International Journal for Quality in Health Care*. 2007. Volume 19, Number 6: pp. 349 – 357.

**Appendix 2.** Interview guide

***Opening statement for the participants:***

Thank you for participating in this study. Before we begin, I’d like to summarize the structure of this session. At first, I’ll be discussing with you about the medications you are currently taking for all your health conditions that may include prescription, non-prescription, and complementary or alternative medicines such as herbal medicines. Following that, I’d like to hear some of your experiences of taking medicines. I also have some questions discussing about your health conditions. These questions will give you an opportunity to talk about your medicines, how well you feel they work, side effects, and so on. This interview should take no more than 30 minutes. Your participation will be completely voluntary and you may prefer not to answer any of the questions if you are not willing to. I’d like to audiotape this conversation for future reference however, all your responses will be kept confidential and will not affect your medical care in any way. Do you have any questions or concerns you’d like to discuss before we begin?

If yes: (give answer)

If no: (begin interview)

***Questions relating to experiences of taking medicines:***

1. How do you **feel about** your medicines?
2. How are your medicines **helping** your illness?
3. How do your medicines **affect your** life?
4. On what way does your medicines affect your **family and social** life?
5. What are the things you **don’t like** about taking your medicines?
6. What is the **most challenging** part of taking your medicines?
7. What **situations** make it difficult for you to take your medicines?
8. What are the things that **helps you** to take your medicines?
9. In what situations you **feel easy** taking your medicines?
10. What are the **problems** you face while taking your medicines?
11. What are the **ways to fix** any of the medication related problems you face?
12. How do you seek help for the **management** of specific symptoms?
13. Where do you go or whom do you **seek for help** to talk about your medicines?
14. When you **see your doctor**, how does the session usually go?
15. What are the **skills** that you have adapted for taking your medicines?
16. How do you manage your **expenses** for medication?

***References:***

1. Ogedegbe G et al. Barriers and facilitators of medication adherence in hypertensive African Americans: a qualitative study. *Ethn Dis*. 2004;14(1):3-12.
2. Williams AF et al. Adherence to multiple prescribed medications in diabetic kidney disease: a qualitative study of consumers’ and health professionals’ perspectives. *Int J Nurs Stud*. 2008;45(12):1742-1756.

**Appendix 3.** Summary of interpretation of themes with exemplar quotes

| **Themes based on WHO taxonomy** | **Exemplar quotes** |
| --- | --- |
| **Patient-related factors^µ^** |  |
| Knowledge and beliefs |  |
| - Lack of understanding about medicines | *“Well, I just don’t know what some of them are for.”* (P1, male, 53 years, PSR NAD) |
|  | *“I don’t know what’s really important and… if you missed [medication] once or twice it wouldn’t matter, I’ve no idea.”* (P5, female, 58 years, PSR NAD) |
|  | *“As far vitamins are no much point for me because it all gets dialysed out of here [pointing to the dialysis machine].”* (P8, male, 71 years, PSR NAD) |
| - Lack of benefit | *“I don’t know if they doing any good? […] I thought well, you know, I am taking all this in the morning, um… are they doing any good? I don’t know.”* (P5, female, 58 years, PSR NAD) |
| - Relative importance | *“I think blood pressure one is important. Yes, I think that is important to keep my blood pressure down…”* (P6, female, 74 years, PSR NAD) |
|  | *“Except for the, ah, Atorvastatin, I’m fairly happy with my medicines.”* (P6, female, 74 years, PSR NAD) |
| - Perceived need | *“I think, you got a put in your head you got a pills because they are trying to help you, so you take them.”* (P11, male, 84 years, PSR AD) |
|  | *“There’s something to do with my kidney and that. […] it’s not working very well. If I started not taking them, I could for been… you know in trouble. They all they are for a reason. Yeah.”* (P15, male, 78 years, PSR AD) |
|  | *“I always take them. If I stop taking them, I don’t do anything, I can’t move. I just stiffen up like this and that’s it.”* (P16, male, 65 years, PSR AD) |
| - Perceived effectiveness | *“I put myself on that [medicine] because I didn’t have any arthritis or anything before I started [dialysis] and all of a sudden my fingers going, and I put it on that now for a month and it stopped the pain…”* (P12, female, 80 years, PSR AD) |
| - Safety concerns | *“I stopped taking them [phosphate binders]. You know, it got me there badly, it got me suffer physically… I mean it must be that one [phosphate binders] because all the other one’s are fine. I haven’t vomited for ages, and I’m very careful about the diet.”* (P5, female, 58 years, PSR NAD) |
|  | *“There’s one medicine that is a statin which I’m very unhappy about. It’s Atorvastatin. And, I’m unhappy about that… because they… they, ah, studies have shown that there are lots of side effects of that.”* (P6, female, 74 years, PSR NAD) |
| Awareness |  |
| - Consequences of nonadherence and motivation to live | *“I don’t know how much longer I got to live. But I want to get up to 80. If I become 80, that will be the longest lived in all our family. And if I make 80… I’m the champion.”* (P15, male, 78 years, PSR AD) |
|  | *“Oh, it [medicines] doesn’t worry me. Its keeping me alive, this medicines keeping me alive so, I do whatever I’ve to. If I don’t take them I’m probably dead.”* (P12, female, 80 years, PSR AD) |
|  | *“If you don’t [take] you won’t breathe.”* (P20, male, 80 years, PSR AD) |
|  | *“doesn’t worry me because they are keeping me alive. Like the treatments keep me alive, the medicines are keeping me alive.”* (P21, male, 84 years, PSR AD) |
|  | *“If I don’t take them I could possibly die. Without having this [dialysis] plus my medication, I wouldn’t last more than two or three weeks.”* (P21, male, 84 years, PSR AD) |
|  | *“Keeps me alive. I want to stay alive. Simple as that.”* (P25, male, 72 years, PSR AD) |
| Attitude |  |
| - Positive attitude | *“I don’t mind taking them [medicines]. It’s better than being, making them worse if you don’t, so.”* (P10, female, 53 years, PSR AD) |
|  | *“They [medicines] are here to be taken, so I take them.”* (P11, male, 84 years, PSR AD) |
|  | *“I always take them, all the time. No matter what, I never stop taking medication. Only what I’ve been prescribed, I don’t take any other medication.”* (P15, male, 78 years, PSR AD) |
|  | *“I got to take them as they keep me healthy. And I don’t have a problem with it.”* (P21, male, 84 years, PSR AD) |
|  | *“[medicines] are to my benefit to take them as prescribed.”* (P21, male, 84 years, PSR AD) |
|  | *“It’s there to take it, you take it. So I don’t have any problem with that.”* (P24, male, 72 years, PSR AD) |
|  | *“They got to take and you take them. Once you start getting sick, they are part of your daily life.”* (P24, male, 72 years, PSR AD) |
|  | *“If they are prescribed for me, I take them.”* (P28, male, 75 years, PSR AD) |
|  | *“You got to take them so you take them… If I don’t have it, I suffer.”* (P28, male, 75 years, PSR AD) |
| - General dislike | *“I don’t like the fact that I need to take them… Not happy about taking medications but the alternatives not good.”* (P13, female, 63 years, PSR NAD) |
| Self-efficacy |  |
| - Disruption to daily routine | *“Well it’s in the morning and night, I’m just used to doing that. It’s the middle one I have to take care of… I take it at night. Take two at night instead of three, spreading three during the day, which the doctor asked me to try, because it might be more effective. I haven’t yet succeeded.”* (P8, male, 71 years, PSR NAD) |
|  | *“I had my wife been on the hospital, and I had been doing things for her and there’s a lot of running around, and just a midday gets left out, so pretty low in the list of priority so, at the moment.”* (P18, male, 71 years, PSR NAD) |
| - Inconvenience during travel | *“When I’m camping, you know there’s a lot to do, it’s just this one extra job in the morning to, you know, wake up in the little tent in the sleeping bag and have to find my pill.”* (P3, male, 44 years, PSR NAD) |
|  | *“People don’t make it difficult for me, but it’s the fact that I’ve, I travel, I like to travel of course make it difficult, because I’ve got to take all the stuffs with me, organize something every day or whatever. Yes, traveling.”* (P6, female, 74 years, PSR NAD) |
| - Accustomed regimen | *“I got all these medications every day, morning, evening, night. So, I never forget it, now.”* (P15, male, 78 years, PSR AD) |
|  | *“Just habit, yeah. In other words, whenever I have my breakfast, my tea, all the time they’re there.”* (P16, male, 65 years, PSR AD) |
|  | *“I have been taking them for a long time, that’s normal for me. Daily routine.”* (P20, male, 80 years, PSR AD) |
|  | *“They are just part of my life. For last 9 years now, I’ve been taking them and I’ve been accustomed to it.”* (P21, male, 84 years, PSR AD) |
|  | *“I’ve been taking it for a long time and it’s just natural.”* (P27, male, 79 years, PSR AD) |
|  | *“I just follow them… [medicines] just normal part of my life.”* (P30, male, 87 years, PSR A) |
| - Unaccustomed regimen | *“I’m supposed to take a medicine for my [restless leg], but I keep forgetting… So, um, I’ve only been told this few days ago and I haven’t got used to it, to taking it.”* (P8, male, 71 years, PSR NAD) |
| Action control |  |
| - Forgetfulness | *“I got some magnesium for cramps, but I forget to take them. I’m also supposed to be taking vitamin D but I hardly ever do. That’s one of those I forget.”* (P8, male, 71 years, PSR NAD) |
|  | *“It’s just that a little forgetful. I’ve put it out at the old age… The only worry is to remember to take them.”* (P14, male, 83 years, PSR NAD) |
|  | *“Well, I think that I’m much more, I don’t know, forgetful then I used to be, I can’t think this clearly, yeah, it’s just a fix with, which seems I pick but I don’t. Um. Remembering to take it. I think that’s the biggest thing.”* (P6, female, 74 years, PSR NAD) |
| - Stimuli or cues for action | *“I have a little pill boxes, it holds all morning, noon and night… I just take whatever is required during dinner, or at meal in the night.”* (P15, male, 78 years, PSR AD) |
|  | *“I have a pill box now. So, I don’t need to worry about remembering. That’s the main issue.”* (P18, male, 71 Years, PSR NAD) |
|  | *“I’ve got a dosette box. It’s got bed time, lunch, and morning”* (P25, male, 72 years, PSR AD) |
| - Visual allocation of pills | *“I’ve got them [medicines] in the kitchen table, so I can’t forget.”* (P10, female, 53 years, PSR AD) |
|  | *“Some of the capsules that I’m on, are on my shelves, taking them all in the morning. On dialysis days, I make sure I leave them and take them when I get home, coz otherwise they just washed forever.”* (P12, female, 80 years, PSR AD) |
| - Association with meals | *“If I don’t have lunch, I don’t remember my medicines, always. Lunch is sort of tied to the medicines. So, if I wouldn’t eat, I wouldn’t take the medicines so regularly, I think.”* (P6, female, 74 years, PSR NAD) |
| Facilitation |  |
| - Role of support | *“I’m retired. I’ve been looked after. Yes, by my daughter. My daughter does all those [medications] for my side and I’ve to put them.”* (P12, female, 80 years, PSR AD) |
|  | *“My wife manages everything. She manages everything. She knows. She’s always done it. Ever since I started taking tablets, she looks after it. She knows what medications, what I’m supposed to do and not supposed to do. You know, she put my tablets at every meal and she’s been doing that for last 14 years. Ever since I had my kidney out.”* (P15, male, 78 years, PSR AD) |
|  | *“If I forget to take them, my wife lets me know… She handles all.”* (P21, male, 84 years, PSR AD) |
|  | *“My wife makes sure I take them... she helps. She gets all medicines ready, tablets ready… she does all, mostly.”* (P27, male, 79 years, PSR AD) |
|  | *“I all live by myself so, just me, I’ve got to worry about my sickness… It’s just me, yeah.”* (P2, male, 61 years, PSR NAD) |
|  | *“Some medicines make me dizzy. It is a problem. Especially when I get no support at home. Coz my husband, he works at night, and I got to be careful. Coz I got no support at home.”* (P7, female, 65 years, PSR NAD) |
| **Health system/ HCT-related factors** |  |
| Quality of interaction with HCT |  |
| - One-way communication | *“[Asking Dr about the need of so many medicines…] I saw doctor at the clinic last time and he said, “No, they are all good”. He went through one by one [medicines] and no, that’s good, you need that, you need that, so…”* (P7, female, 65 years, PSR NAD) |
| - Lack of engagement | *“[Consultations are] never very long usually, you know. Just checks the figures, just look at your blood figures and everything’s ok and you know.”* (P2, male, 61 years, PSR NAD) |
|  | *“Not usually. Unless I’ve a particular problem like my Gout is worse or I’m feeling more depressed. Um, otherwise no. it’s [consultation] all very routine.”* (P4, male, 56 years, PSR NAD) |
| - Lack of time | *“I really need to speak to the pharmacist. Um, but they’re very busy, but I will, I must speak to, I want to know what every medicines, especially 12 medicines in the morning are for.”* (P5, female, 58 years, PSR NAD) |
| - Support from HCT | *“You know, just, give all your tablets to the chemist and he’ll sort them out. Makes it so much easier. Coz, he puts them in a pack, a plastic bag [Webster-Pak], um, and he get it for two weeks and you got a just twist and pop a tablets, all those ones you gonna take, so I don’t need to get worry about what one of this, one of this, anymore.”* (P16, male, 65 years, PSR AD) |
|  | *“It’s always great with my GP. I’ve been going to him for 15 years and we’re quite informal and he’s very helpful and if I complained about what these things, he investigates them properly.”* (P11, male, 84 years, PSR AD) |
| Mistrust and collateral arrangements |  |
| - Pressure to hide | *“I forgot to say to him [doctor] about it [not taking phosphate binders]. Because, I think what they will gonna tell me is, I have to take it. I’m frightened obvious the doctor’s gonna say, which they probably will, because it’s very important, the phosphate, I know that.”* (P5, female, 58 years, PSR NAD) |
|  | *“I did say the kidney doctor months ago, if they [medicines] were helping remove the fluid, um, because I still have a lot of fluid, and he said just keep taking them, you know, and don’t worry about that, you need to keep taking them so, even if it helps a little bit.”* (P5, female, 58 years, PSR NAD) |
| - Being a good patient | *“I just take them because, that I’m following the doctor’s instructions, I don’t… Well, he has his own agenda and he usually takes control of the situation, all them.”* (P6, female, 74 years, PSR NAD) |
|  | *“I don’t. I don’t know I take it because I’ve been told to take it, and I do that. But I don’t take it very seriously. And if I miss it, I don’t get panic, so.”* (P8, male, 71 years, PSR NAD) |
| - Personal control of treatment | *“I used to be on Lipitor and stuffs like that, now I don’t use them. I don’t think I agree with the doctor… I didn’t like those… had made me problems… also doctors don’t put me on Magnesium, I just put myself on it.”* (P2, male, 61 years, PSR NAD) |
|  | *“I discuss it with myself. Or, I go to them [doctor] who gets upset because I decide to take more than what I’m prescribed. Yeah, like the Sifrol, it wasn’t holding, so I lifted the thing [dose] up to two. And I checked it out [in the internet] and it was okay to do that and then she [doctor] got most upset because she said it effects the kidney, and I said well they’re pretty shot already, and she said they can always get worse.”* (P8, male, 71 years, PSR NAD) |
|  | *“Because the doses are too, too [high]…, they have got to decrease it. Coz, I’m taking one tablet, and then they took me off and put it on the other one, and the other one they put me on was too high. Makes me dizzy. So I didn’t take anymore.”* (P7, female, 65 years, PSR NAD) |
|  | *“I’m supposed to take it [blood pressure medicine] every day but, I’ve been taking it every second or third day because of the, coz my blood pressure really low. So, so far I’ve been able to control at that way. So I decided to stop, if I can do without it, I will… I don’t take it terribly seriously.”* (P8, male, 71 years, PSR NAD) |
| - Trust in HCT | *“I take my medicines. They give me the right thing, so I just take them. Except when I’m allergic to.”* (P10, female, 53 years, PSR AD) |
|  | *“I’m consistent about it. Because I’ve got to take them daily or as prescribed, so I always do as I’m told.”* (P11, male, 84 years, PSR AD) |
|  | *“My doctor is a gentleman and the scholar. He is in charge of it, and he put me in these medication, I take.”* (P15, male, 78 years, PSR AD) |
|  | *“That’s what doctor prescribes and I take them.”* (P20, male, 80 years, PSR AD) |
|  | *“I keep taking them until my doctor takes me out of it. I just take the dose that’s on the charts I got.”* (P25, male, 72 years, PSR AD) |
| **Therapy-related factors** |  |
| Physical characteristics of medicines |  |
| - Pill size | *“I’ve got the one [medicine], got to cut it half, I’ve got a cut five or six in half so I’ve got half for in the morning and half at night.”* (P9, female, 63 years, PSR NAD) |
|  | *“I can’t take the big ones. The size of the [phosphate binders], sometimes I vomit in backyard. I can’t handle big tablets. Large tablet size, too hard, yes, to swallow. Yeah.”* (P10, female, 53 years, PSR AD) |
| - Palatability | *“Some of them, as soon as you get them on the tongue, I think that, why not take… I swear it, dissolves straight away and it tastes disgusting! First thing in the morning they, oh! You know, then try to get the water, buff! Just bitter, you know, one of them.”* (P5, female, 58 years, PSR NAD) |
|  | *“Apart from anything else, some of them taste absolutely disgusting… especially, when the ones like the Allopurinol where you’ve got them to cut in half, they taste pretty disgusting.”* (P13, female, 63 years, PSR NAD) |
|  | *“There was one tablet that taste like a lolly, and now they don’t. They have changed the medicine.”* (P22, male, 65 years, PSR NAD) |
| Medicine packaging | *“One I have very hard to get it out. A little capsule, that for pain. Yeah. Very hard to put out. The capsules are completely crushed by the time it gets out of its thing! That’s the only problem.”* (P11, male, 84 years, PSR AD) |
| Side effects of medicines | *“Sometimes they work, sometimes they really make me sick. Makes me dizzy. Coz it’s a bit stronger. I don’t take them. Well, if they are not too strong, I’ll take them. I always take my medicines, but if they will make me dizzy, I don’t.”* (P7, female, 65 years, PSR NAD) |
|  | *“I know, when I don’t take them, I feel better. That feel so severe, vomiting and nausea… so, when I was taking [phosphate binders] it was quite severe… so, I thought no to horrible thing!”* (P5, female, 58 years, PSR NAD) |
|  | *“I don’t like taking them, the [antibiotics], they give me toilet all the time.”* (P29, male, 65 years, PSR NAD) |
| **Social/ economic factors** |  |
| Access to medicines |  |
| - Acquiring script | *“I was hoping if someone can bring those [scripts] here at dialysis unit, so I can just pick them up, when I’m on dialysis. It would be lot easier.”* (P2, male, 61 years, PSR NAD) |
|  | *“The only hassle is, as far as [blood pressure medicine] is concerned, its supply is only 25 days, so I have to keep asking for repeat.”* (P23, male, 86 years, PSR NAD) |
|  | *“I’m taking a lot of pain tablets at the moment. Finger’s pain at all time. I was taking patches, but you can’t get it more than a month’s supply. So, that means going back on doctors, and when I get out of here [dialysis], I don’t want have to go waiting, in a waiting room to get to the doctors on my days off [from dialysis], so I’m just taking Panadol and Panadol with Codeine. But, is not really enough, to be honest.”* (P2, male, 61 years, PSR NAD) |
| - Clinic and pharmacy location | *“Because I live out of town, I live an hour out of Hobart, and about 40 minutes from the chemist, just kind of be aware how many more medicines I’ve got, it’s nothing worse than running out and having to drive especially for that, yeah.”* (P3, male, 44 years, PSR NAD) |
|  | *“Some of the scripts you can’t get from pharmacy [local pharmacy]. So, I’ve had issues actually getting them in the past… When my doctor goes on holidays, I can’t acquire a script without doing it a 100 km drive. They [dialysis staffs] refused to help me, and the public doctors refused to give me scripts over the phone. I can’t acquire a script over the phone. So I got a drive, do a 100 km drive just to get a script from the public doctors.”* (P1, male, 53 years, PSR NAD) |
| Relative affordability | *“Well, they’re quite expensive! So they do affect me, the cost. I don’t have a health care card. I’ve to pay the full subsidised price… I’ve retired and so I’m living of an allocated pension from my superannuation.”* (P4, male, 56 years, PSR NAD) |
|  | *“The only thing that worries me is, coz I’m in a wheel chair and I need to get to the hospital to get the scripts, it means for $ 30 to get in the taxi to go in there and pick up the script or I drive my mobility scooter all the way in there, which means two hours and an hour of each waiting to pick them up.”* (P2, male, 61 years, PSR NAD) |
|  | *“It’s mainly because it costs me a lot of money every month… it is very expensive… I think I take 12 [medications]. It’s mainly the expense of the medications. I added it up the other day, for one month... Sixty-a-dollars, sometimes it varies.”* (P5, female, 58 years, PSR NAD) |
| **Condition-related factors** |  |
| Symptom severity | *“Have you seen me 12 months ago, I am on a 100 % better [condition] after this year but last year and a year before, no, I didn’t really think I’m gonna make it. Not even everybody else also gonna make it either.”* (P12, female, 80 years, PSR AD) |
|  | *“I don’t notice any [improvement] from my medications, whatsoever.”* (P1, male, 53 years, PSR NAD) |

*Abbreviations:* AD, Adherent; NAD, nonadherent; HCT, healthcare team; PSR, patient self-reports

^µ^ Patient-related factors further classified based on adherence support taxonomy by de Bruin *et al*., 2010.
